# A mechanistic approach to anti-nociceptive potential of *Artemisia macrocephala J*acquem

**DOI:** 10.1186/s12906-016-1114-0

**Published:** 2016-05-26

**Authors:** Mohammad Shoaib, Ismail Shah, Niaz Ali, Wadood Ali Shah

**Affiliations:** Department of Pharmacy, University of Malakand, Chakdara Dir Lower, Khyber Pakhtunkhwa (KPK) Pakistan; Institute of Basic Medical Sciences, Khyber Medical University Peshawar, Peshawar, KPK Pakistan

**Keywords:** *Artemisia macrocephala*, Acute toxicity, Antinociceptive activity, Formalin, Acetic acid, Tail immersion

## Abstract

**Background:**

*Artemisia macrocephala* Jacquem (*A. macrocephala*), locally known as “Tarkha”, is a perennial plant found abundantly in northern areas of Pakistan. It is widely used in traditional medicine to treat fever, pain, gastrointestinal disorders and diabetes. Till date, no published studies are available regarding the in-vivo antinociceptive potential of the crude extract and sub-fractions from the aerial parts of *A. macrocephala*.

**Methods:**

Antinociceptive effects of the crude methanolic extract and its sub-fractions were assessed using experimental pain models, including chemical nociception induced by intraperitoneal acetic acid or subplantar formalin injection and thermal nociception like tail immersion test in-vivo.

**Results:**

The administration of various doses of crude extract and its fractions showed a dose-dependent indomethacin like antinociceptive effect in acetic acid induced writhing, subplantar formalin injection animal model suggesting the involvement of central mechanism of pain inhibition. Moreover, the crude extract and sub-fractions, on tail flick model (thermal nociception) demonstrated the involvement of central mechanism and significantly increased the latency time to 66.54, 82.94 and 70.53 %. The antagonistic study proposed the possible involvement of opioid receptor using naloxone as non-selective antagonist. The pharmacologically active chloroform and ethyl acetate fractions were further subjected to column chromatography that lead to the isolation four compounds. These isolated compounds were then subjected to various spectroscopic techniques upon which they were confirmed to be one sterol and three flavonoid derivatives. These findings suggest that *Artemisia macrocephala* possesses peripheral and central analgesic potentials partially associated with opioid system that support its folkloric use for the management of pain. The isolated compounds are currently under investigation in our laboratory for analgesic activity and its possible mechanism of action.

**Conclusion:**

The results in this study provide evidences that *A. macrocrphala* has anticonciceptive effects and can be used for treatment of pain in traditional therapies. This study opens a new channel for isolation of analgesic compounds from the specie that is used traditionally for the management of pain.

**Electronic supplementary material:**

The online version of this article (doi:10.1186/s12906-016-1114-0) contains supplementary material, which is available to authorized users.

## Background

Despite of the present scientific advancement in pain therapies, potent, safe and effective drugs are still needed for many painful conditions [1]. The current available therapies for pain, mostly elicit severe adverse effects [2, 3]. Therefore, drugs with no such effects are searched throughout the world [4]. Natural products like plants, animals, microbes and marine life have contributed to almost 50 % of current drugs that are used as a significant tool for the management of various ailments [5]. Searches from the past have led to the discovery of active analgesic from plants in shape of aspirin and morphine [6, 7].

The genus *Artemisia* has diverse uses in traditional system of medicine. Its members are used for abdominal pains and colds in the head, as expectorant for bronchitis and asthma, as poultice for rheumatoid arthritis, and as a plaster cast for broken bones [8]. Scientifically, many of its plants had been reported to possess anti-inflammatory, insecticidal, analgesic, antispasmodic, antifungal, antibacterial and photo protective [9], while some of its species are used during labor pain [10]. On scientific basis, *Artemisia herba alba* had been proved to have clear antinociceptive and anti-inflammatory effects [11], *Artemisia copa* presents antinociceptive and topical anti-inflammatory [12], *Artemisia absinthium* possesses topical analgesic [13] and *Artemisia dracunculus* has significant analgesic and anti-inflammatory effects [14]. Different classes of chemicals, e.g., coumarins, terpenes, sesquiterpenes, flavonoids, aromatics, dipeptides, phenolics, coumarins, esterols, germacranolides, guaianolides, secoguaianolides and polysaccharides have been isolated from *Artemisia* species [15]. These constituents impart a wide-range of pharmacological effects like analgesic [16–19] antitumoral, antioxidant, anti-inflammatory [17–19] anti-cancer and immunomodulatory action [20–23]. Some species of this genus are used in beverages and in infusions for their digestive properties, serves as a base for some bitter drinks while its oils possess trypanocydal and mosquito repellent properties [24]. *A. macrocephala* Jacquem is an important member of this genus which is found abundantly in Northern Areas of Pakistan [25]. The preliminary phytochemical investigations confirmed the presence of terpenes, flavonoids, saponins and alkaloids [26]. Our previous work on this specie revealed its traditional use for the management of gut spasms and confirmed its antispasmodic response using in vitro rabbits’ jejunal preparations. Apart from that, it also possesses antioxidant activity [25]. In another study, our group worked on its enzyme inhabiting potentials and confirmed that its essential oil obtained by process of hydro distillation has significant enzyme inhibition activity against acetylcholinesterase and butyrylcholinesterase activity that may be a possible alternative for treating Alzheimer’s disease [27]. Apart from other folkloric uses, this specie is known for its anti-inflammatory and analgesic response; therefore, the present study was designed to explore on scientific basis the traditional use of *A.macrocephala* for possible antinociceptive potential using animal model. The finding of this study may further lead to the investigation for the isolation and structure elucidation of bioactive compounds responsible for its anti-inflammatory and analgesic activities.

## Methods

### Collection and authentication of plant materials

Fresh aerial parts of *A. macrocephala* were collected in August, 2014 from the nearby hills of Badwan Chowk, Dir Lower, Khyber Pakhtunkhwa, Pakistan. The species was identified by plant taxonomist, Dr. Nasur Ullah, Assistant Professor University of Malakand Chakdara Dir Lower Khyber Pakhtunkhwa, Pakistan. A voucher specimen “Am-01–2014” was submitted to the herbarium of Department of Botany, University of Malakand.

### Extraction

The plant material was kept in shade at room temperature for drying. It was afterwards crushed and pulverized by a mechanical grinder to obtain fine powder. The fine powder (5.5 kg) was then macerated in commercial grade methanol with occasional shaking for 22 days at room temperature. The materials were then filtered off. The process was repeated 3 times. The filtrates were combined and evaporated under reduced pressure, using a rotary evaporator, until a dark greenish color crudemethanolic extract (Am.Cme) was obtained.

### Fractionation

The crude Am.Cme. (500 g, 9.09 %) was suspended in distilled water (400 mL), which was successively fractionated with 400 mL of *n*-hexane. The process was repeated until complete fractionation. Similar procedure was followed for fractionation with chloroform and ethyl acetate till it successively gave 44 g of *n*-hexane fraction (Am.*n*-hex, 8.8 %), 77 g of chloroform fraction (Am.Chf, 15.4 %), 40.4 g of ethyl acetate (Am.EtOAc, 8.08 %) and 32 g of residual aqueous fraction (Am.Aq, 6.4 %). The fractions were refrigerated till pharmacological screenings.

### Preliminary phytochemical screening and isolation of compounds

Based upon our previous findings of various phytochemicals in the crude extract [25], the subsequent fractions were subjected to preliminary phytochemical screening to explore the natural product rich fraction. Further, the pharmacologically active fractions (chloroform and ethyl acetate) were subjected to column chromatography for isolation of active chemical constituents that were further subjected to various spectroscopic techniques like mass, 1H-NMR, NOESY, COSY, HMBC and HSQC for structure elucidation.

### Animals and ethical approval

Animals used, were Swiss albino mice of either sex. They were obtained from animal house of Department of Pharmacy, University of Malakand, Chakdara, Dir Lower, KPK, Pakistan. They were maintained under standard conditions of laboratory and were fed standard food and water *ad libitum*. All the protocols of the experiment were approved by ethical committee of Department of Pharmacy (No: E-AM-01–2009), University of Malakand as per Bye Laws 2008 of the University of Malakand (Scientific Procedures Issue-I).

### Acute toxicity

The crude methanolic extract and fractions were tested for acute toxicity study as per standard protocol [28]. Swiss albino mice were administered 500, 1000, 1500 and 2000 mg/kg of the crude extract and fractions per oral route. One group received normal saline that served as negative control. The animals were observed for 6 h continuously for changes in their behavior. Mortality for the next 14 days was also noted.

### Analgesic activity

#### Acetic acid induced writhing test

The peripheral antinociceptive response is determined through acetic acid induced writhing test.

Two groups of mice (*n* = 6) for each sample were prepared. They were given 150 and 300 mg/kg of crude extract and 100 and 200 mg/kg (orally) of sub-fractions 1 h before the injection of 10 mL/kg of 1 % acetic acid intraperitoneally. Negative control group was given 10 mL/kg of 1 % solution of Tween 80 (1 %, v/v) while positive control group was given 50 mg/kg of diclofenac sodium intraperitoneally to overnight fasting mice. Writhing and stretching number was noted and percent protection was determined from the data [29].

#### Formalin test

This test was carried out as per Pandurangan et al. [30] protocol with a bit modification. Five mice per group were given different doses (150 and 300 mg/kg crude and 100 and 200 mg/kg sub-fraction) of test samples. After 30 min of administration of test samples, 2.5 % formalin (20 μl) was injected subcutaneously (s.c) in mice’ hind paw. The time spent in licking of the test paw (to which test samples were injected) in early phase (0–5 min) and late phase (15–30 min) were recorded. Naloxone, opioid antagonist (2 mg/kg, s.c), standard drug indomethacin (10 mg/kg) and Tween 80 (2 %, v/v) were also used.

#### Tail immersion test

In order to determine the central antinociceptive response of the test samples, the animals were given different doses (150 and 300 mg/kg crude and 100 and 200 mg/kg sub-fraction) of the sample intraperitoneally, 2 % vehicle and 50 mg/kg of diclofenac sodium, 30 min prior to the immersion of the tail (3 cm) into hot water (55 ± 0.5 °C). To assess the possible involvement of opioid receptors, morphine (agonist) in a dose of 5.0 mg and naloxone (antagonist) in a dose of 2.0 mg were used. The time of reaction taken at 15, 30, 45, 60, 75 and 90 min after administration of test samples were noted using a stopwatch [31].

#### Statistical analysis

Data are presented as mean ± SEM (*n* = 6). Graph Pad Prism was used for analysis of data followed by Dunnett’s test for statistical significance using *p* ≤ 0.05.

## Results

Results for the preliminary phytochemical screening reveal the presence of flavonoids and saponins in the chloroform fraction and sterol and flavonoid in the ethyl acetate fraction. The chloroform fraction was found to be rich with flavonoids. Apart from flavonoids, saponins and terpenes were also present but in mild quantity. The pharmacologically active fractions (chloroform and ethyl acetate) lead to the isolation of four compounds. The integration of the isolated compounds Ism-1 to Ism-4 are summarized as under, while the original spectra are given in Additional file 1.

### Ism-1

Pale yellow amorphous powder, IR (KBr, Cm^−1^), 3391, 3263, 1659, 1519. UV λ_MeOH_ max nm: 342 and 347; ^1^H-NMR (500 MHz, CD_3_OD): δ 7.59 (d, 1H, *J*_6′,5′_ = 8.0 Hz, H-6′), 7.10 (br s, 1H, H-2′), 7.09 (d, 1H, *J*_5′,6′_ = 8.0 Hz, H-5′), 6.63 (s, 1H, H-2), 6.52 (s, 1H, H-8), 3.92 (s, 3H, H-4′/OCH_3_), 3.90 (s, 3H, H-3′/OCH_3_), 3.86 (s, 3H, H-6/OCH_3_), δ EI-MS: *m/z* 344 (M^+^), 329, 326, 301 [32].

### Ism-2

Light yellow amorphous powder, 1H-NMR (500 MHz, acetone-*d*_6_) δ 12.98 (s, 1H, H-5/OH), 7.96 (d, 2H, *J*_2′,3′/6′,5′_ = 8.0 Hz, H-2/H-6), 7.03 (d, 2H, *J*_3′,2′/5′,6′_ = 8.0 Hz, H-3/H-5), 6.68 (s, 1H, H-3), 6.66 (d, 1H, *J*_8,6_ = 2.0 Hz, H-8), 6.32 (d, 1H, *J*_6,8_ = 2.0 Hz, H-6), 3.92 (s, 3H, H-4′/OCH_3_). EI MS *m/z* (%) 284 (M^+^), 255 [33].

### Ism-3

Slight yellow powder, IR (KBr, Cm^−1^), 3500, 1662, 1614, 1512. UV (AlCl_3_ + MeOH): λmax: 484 (2.86), 272 (2.79) and 1H-NMR (500 MHz, MeOD) δ 6.18 (1H, *d, J* = 2.0 Hz, H-6), 6.39 (1H, *d, J* = 2.0 Hz, H-8), 6.88 (1H, *d, J* = 8.3 Hz, H-5′), 7.62 (1H, *dd*, *J* = 8.3; 2.1 Hz, H-6′), 7.74 (1H, *d*, *J* = 2.1 Hz, H-2′, ESI-MS: *m/z* 300.9 [M-H]-, 602.6 [2 M-H] [34].

### Ism-4

White, waxy powder, IR (KBr, Cm^−1^), 3373.6, 2940.7, 2867.9, 1641.6, 1457.3, 1381.6, 1038.7, 881.6. UV λ CHCl_3_ max nm: 220. 1H-NMR (500 MHz, CDCl3) δ (ppm) = 0.74 (1H, *s*, H-18), 0.87 (1H, *d*, *J* = 6.9 Hz, H-27), 0.88 (1H, *d*, *J* = 6.9 Hz, H-26), 0.89 (1H, *t*, *J* = 7.4 Hz, H-29), 0.93 (1H, *d*, *J* = 6.5 Hz, H-21), 0.97 (1H, *m*, H-24), 0.98 (1H, *m*, H-9), 1.04 (1H, *m*, H-14), 1.06 (1H, *s*, H-19), 1.07 (1H, *m*, H-22b), 1.11 (1H, *tm*, *J* = 11.2 Hz, H-15b), 1.13 (1H, *m*, H-1b), 1.16 (1H, *t*, *J* = 10.0 Hz, H-17), 1.21 (1H, *m*, H-23), 1.21 (1H, *m*, H-12b), 1.30 (1H, *m*, H-16b), 1.31 (1H, *m*, H-28), 1.36 (1H, *m*, H-22a), 1.40 (1H, *m*, H-20), 1.50 (1H, *qd*, *J* = 10.8; 4.6 Hz, H-11b), 1.50 (1H, *m*, H-7), 1.55 (1H, *m*, H-11a), 1.56 (1H, *m*, H-2b), 1.63 (1H, *m*, H-15a), 1.71 (1H, *m*, H-25), 1.88 (1H, *m*, H-2a), 1.89 (1H, *m*, H-16a), 1.90 (1H, *m*, H-1a), 2.03 (1H, *td*, *J* = 12.1; 2.4 Hz, H-8), 2.06 (1H, *dt*, *J* = 12.8; 3.6 Hz, H-12a), 2.30 (1H, *td*, *J* = 11.0; 2.0 Hz, H-4b), 2.34 (1H, *ddd*, *J* = 13.0; 5.0; 2.0 Hz, H-4a), 3.58 (1H, *tt*, *J* = 11.3; 5.3 Hz, H- 3), 5.40 (1H, *dd*, *J* = 5.2; 2.3 Hz, H-6); ESI-MS: *m/z*:437 [M + Na] + [35].

The crude methanolic extract and sub-fractions in test doses of 500, 1000, 1500 and 2000 mg/kg body weight showed no adverse effects on the behavioral responses in the tested mice following 14 days observation. No mortality or weight change observed. Therefore, a highest dose of 300 mg/kg given to mice in this study was considered to be safe.

In performing the acetic acid induced writhing test for the determination of antinociceptive effect, the samples showed a significant analgesic effect. The crude methanolic extract, chloroform and ethyl acetate fraction showed a dose dependent response and significantly inhibited the acetic acid induced writhings with a maximum value of 62.64 % (*P* < 0.01, *n* = 6), 71.88 % (*P* < 0.001, *n* = 6) and 66.93 % (*P* < 0.001, *n* = 6), respectively as shown in Table 1. Whereas, the residual aqueous fraction showed no significant activity (data not shown). All the results were compared against standard (diclofenac sodium, 50 mg/kg) with 84.96 % response.

**Table 1:** Acetic acid induced analgesic activity of test samples of *Artemisia macrocephala*

Treatment/dose	Number of writhings	% inhibition
Control (2 % Tween 80)	67.59 ± 1.02	---
Crd 150 mg 300 mg	31.63±1.25**	53.20
	25.25±1.05**	62.64
Chf 100 mg 200 mg	27.25±1.20**	59.68
	19.00 ± 1.35***	71.88
EtOA 100 mg 200 mg	28.45±1.15**	57.90
	22.35±1.20**	66.93
Diclofenac sodium (50 mg)	10.16±0.70***	84.96

All the values were expressed as mean ±SEM (*n*=6). **P<0.01, ***P<0.001 when compared to control group

*Key: Crd* crude methanolic extract, chf chloroform fraction, *EtOA* ethyl acetate fraction

After the administration of the crude extract and its sub-fractions to the animals treated with formalin, it showed a dose dependent response and significantly inhibited both the phases with 37.93 % (^**^P < 0.01, *n* = 6), 51.34 % (^***^P < 0.001, *n* = 6) and 45.33 (^**^P < 0.01, *n* = 6) in the first phase and 56.87 % (^***^P < 0.001, *n* = 6), 73.54 % (^***^P < 0.001, *n* = 6) and 66.63 % (^**^P < 0.01, *n* = 6) of crude methanolic extract (300 mg/kg), chloroform and ethyl acetate fraction (200 mg/kg) respectively, for the second phase. While the aqueous fraction showed no any prominent response (data not shown).

Animals treated with morphine (5.0 mg) significantly inhibited both the phases with 86.86 % (^***^P < 0.001, *n* = 6) and 96.11 % (^***^P < 0.001, *n* = 6) for first phase and second phase respectively as shown in Table 2. A reversal of inhibitory potential was observed in animals pre-treated with naloxone.

**Table 2:** To show effects of *Artemisia macrocephala* on formalin-induced paw-licking response

Treatment/dose		Licking	Time (sec)	Inhibition (%)	
		1st phase	2nd phase	1st phase	2nd phase
Control (2 % Tween 80)		50.03 ± 1.63	72.00 ± 1.30	----	
Crd	150 mg	34.22±1.12**	37.25±1.619**	31.60	48.26
	300 mg	31.05±1.125**	31.05±1.668***	37.93	56.87
Chf	100 mg	28.25±1.65**	30.50±1.425**	43.53	57.63
	200 mg	24.34±1.411***	19.05±1.039***	51.34	73.54
EtOA	100 mg	28.96±1.55**	30.95±1.441***	42.11	57.01
	200 mg	27.35 ±1.05**	24.02 ±0.95**	45.33	66.63
Indomethacin (10 mg)		39.83±1.55**	18.66±1.542***	20.38	74.08
Morphine (5 mg)		6.41±1.165***	2.83±1.260***	86.86	96.11
N + Crd	150 mg	47.15±1.75	63.60±1.542	5.75	11.66
	300 mg	47.70±1.50	64.50±1.428	4.65	10.41
N + Chf	100 mg	47.80±1.25	65.35±1.30	4.45	9.30
	200 mg	48.05±1.33	66.65±1.45	3.95	7.43
N + EtOA	100 mg	47.75±1.23	64.85±1.40	4.55	9.93
	200 mg	47.95±1.25	65.95±1.416	4.15	8.40
N+ Indomethacin (10 mg)		44.00±1.84**	25.00 ±1.539***	12.05	65.27
N + Morphine (5 mg)		47.66±1.52	71.83±1.142	2.39	1.37

All the values were expressed as mean ±SEM. *P<0.05, **P<0.01 and ***P<0.001 whencompared to control group (one way ANOVA followed by Dunnett’s: compare all vscontrol test)

*Key: Crd* crude methanolic extract, *chf* chloroform fraction, *EtOA* ethyl acetate fraction, *N* naloxone 2 mg

The results show that naloxone caused a prominent reversal effect of the analgesic potential of morphine in both the phases. Indomethacin (10 mg/kg) mildly inhibited the first phase with 20.38 % (^***^P < 0.001, *n* = 6) and significantly inhibited the second phase with 74.08 % (^***^P < 0.001, *n* = 6).

The samples (crude methanolic extract 300 mg/kg, chloroform 200 mg/kg and ethyl acetate fraction 200 mg/kg) when tested for its effectiveness in tail flick model, significantly increased the latency time to 66.54 % (^**^P < 0.01, *n* = 6), 82.94 % (^**^P < 0.01, *n* = 6) and 70.53 % (^***^P < 0.001, *n* = 6) respectively at 60 min at which morphine (opioid analgesic, centrally acting), showed 85.07 % (^***^P < 0.001, *n* = 6) activity resembling more to that of the chloroform fraction. Naloxone treated animals significantly reduced the analgesic potentials of morphine and the test samples (Table 3).

**Table 3:** To show analgesic activity of *Artemisia macrocephala* (tail flick method) and standard

Treatment/dose	Time in sec (tail flick)/response (%)					
	15 min	30 min	45 min	60 min	75 min	90 min
Control (2 % Tween 80)	0.79±0.035	0.89±0.025	0.97±0.027	0.94±0.036	0.87±0.027	0.93±0.045
Crd 150 mg 300 mg	0.97±0.130 (18.55 %)	1.24±0.124* (28.22 %)	1.69±0.139 (42.60 %)	2.10±0.122** (55.23 %)	2.34±0.134** (62.82 %)	2.05±0.124** (54.63 %)
	1.09±0.130* (27.52 %)	1.40±0.121* (36.42 %)	1.92±0.129** (49.47 %)	2.77±0.130** (66.06 %)	3.18±0.165*** (72.64 %)	3.03±0.140*** (69.30 %)
Chf 100 mg 200 mg	1.10±0.995* (28.18 %)	1.35±0.399* (34.07 %)	1.98±0.236** (51.01 %)	3.37±0.220** (72.10 %)	3.75±0.213*** (76.80 %)	3.31±0.237*** (71.90 %)
	1.16±0.029* (31.89 %)	1.41±0.021* (36.87 %)	2.10±0.042** (53.80 %)	5.29±0.060** (82.23 %)	4.33±0.041*** (79.90 %)	4.47±0.057*** (79.19 %)
EtOA 100 mg 200 mg	1.04±0.214* (24.03 %)	1.29±0.241* (31.00 %)	1.87±0.361** (48.12 %)	3.10±0.421** (69.67 %)	3.38±0.302** (74.26 %)	2.76±0.280*** (66.30 %)
	1.10±0.201* (28.18 %)	1.33±0.202* (33.08 %)	1.91±0.350** (49.21 %)	3.19±0.435** (70.53 %)	3.30±0.313*** (73.63 %)	2.79±0.291*** (66.66 %)
Standard (Morphine 5 mg)	1.52 ±0.024 (48.02 %)	2.03 ±0.066 (56.15 %)	3.89±0.038 (75.06 %)	6.30±0.054 (85.07 %)	4.40±0.050 (80.22 %)	4.30±0.074 (78.37 %)
N +Crd 150 mg 300 mg	0.79±0 .1 0 8	0 .95±0 .044	0 . 99±0.045	0.99±0.064	0.95±0.026	0.81±0.042
	0.84±0.046	0.98±0.031	1.14±0.047	0.99±0.035	0.96±0.051	0.92±0.034
N +Chf 100 mg 200 mg	0.93±0.048	0.92±0.036	1.10±0.031	1.10±0.047	0.90±0.038	0.93±0.061
	0.87±0.037	0.91±0.065	1.50±0.049	0.99±0.038	0.89±0.029	0.95±0.044
N +EtOA 100 mg 200 mg	0 . 89±0.03 3	0 .93±0 .0 5 1	1.20±0.051	1.11±0.034	0.91±0.061	0.90±0.056
	0.85±0.037	0.90±0.065	0.99±0.049	1.12±0.038	0.91±0.029	0.99±0.044
Morphine (5 mg) + Naloxone (2 mg)	0.77±0.022	0.87±0.036	0.94±0.040	0.91±0.034	0.91±0.030	0.91±0.042

All the values were expressed as mean ±SEM. *P<0.05, **P<0.01 and ***P<0.001 when compared to control group (one way ANOVA followed by Dunnett’s: compared all vs control test)

*Key: Crd* crude methanolic extract, *chf* chloroform fraction, *EtOA* ethyl acetate fraction, *N* naloxone 2 mg

## Discussion

There are many natural products that are used in traditional medicines in various countries. Alternative system of medicine for various ailments is receiving much more attention. Many plants produce significant effect(s) like synthetic drug(s) [36]. Therefore, natural products with little side effects are compulsory to take place of chemical therapeutics [37]. Data from the literature showed that some species of the genera *Artemisia* possess analgesic activity and these pharmacological effects have been attributed mainly to flavonoids, alkaloids, sesquiterpene lactones and essential oils [38].

The main focus of our work is to confirm on scientific basis that *A. macrocephala* possesses antinociceptive activity. This conclusion is strengthened by three different methods used for the evaluation of antinociceptive activity. The results obtained from this study showed the presence of antinociceptive potentials of *A. macrocephala* in all animal models used. Acetic acid induced abdominal constriction is a quite sensitive practice that enables the detection of peripheral antinociceptive activity [9]. This test causes localized inflammation in mice due to biosynthesis of prostaglandins and leukotriens through cyclooxygenase and lipooxygenase pathway [39]. This model is based on several nociceptive mechanisms, like sympathetic system (biogenic amines release), cyclooxygenases and their metabolites [40] and opioid mechanisms [41]. Therefore, the formalin and tail immersion tests were used to find out if the plant possesses any central analgesic potential.

The formalin test is considered a valid model for clinical pain [42] and in this model; the crude extract and sub-fractions effectively inhibited the licking response in both early and late phases, in a manner more similar to that of morphine. An opioid antagonist, naloxone, greatly reversed the analgesic effect of the crude extract and sub-fractions indicating that opioid receptors are involved in the activity. However, complete antagonism was not observed, therefore, it may be concluded that the effect was due to flavonoids [43], as the plant tested positive for the presence of flavonoids [25].

In tail flick test the crude extract and sub-fractions again showed morphine like response indicating that the crude extract and sub-fractions have spinal effect. NSAIDs (Non-steroidal anti-inflammatory drugs) act by decreasing the sensitivity of pain receptors which is caused by prostaglandins [44]. Though the exact mechanism is not known, the observed activity may be due to morphine like effect of the samples. A lot of substances with spinal analgesic activity are present in literature among which the most familiar one is morphine [45]. In a similar way, we tried to find out on scientific basis the antinociceptive potentials of *A. macrocephala,* which showed morphine like antinociceptive activity, which increases the list of pharmacological activities already mentioned for *A. macrocephala*. This discovery encourages our previous work of relaxant activity of the crude extract and essential oil of *A. macrocephala* [25]. Moreover, flavonoids are polyphenolic compounds and are plant’s secondary metabolites. They are becoming the focus of medical research since they possess useful properties like antiviral [46], anti-inflammatory, antitumour, antiallergic, antimicrobial, antioxidant, cytotoxic, enzyme inhibition, oestrogenic [47, 48], vasodilating [49], antipyretic and analgesic activities [50]. Based upon the above findings (Tables 1, 2 and 3), and the pharmacological potentials of flavonoids, the pharmacologically rich chloroform and ethyl acetate fractions were subjected to column chromatography for the isolation of active bio molecules. These bio-molecules (Ism-1, Ism-2, Ism-3 and Ism-4) are currently under scientific investigations for possible analgesic activity and its mechanistic approach to determine the involvement of various receptors apart from opioid receptor that play important role in nociception. This ongoing research will standardize the specie for bio-guided isolation of targeted pharmacologically active moieties.

## Conclusion

The results show that *A. macrocephala* is rich with bioactive compounds producing analgesic effect. *A. macrocephala* can be further investigated for the isolation and structure elucidation of the bioactive compounds responsible for analgesic activity and to explore its possible mechanism of action.

## Additional file

Additional file 1: Spectra analysis. (DOC 6349 kb)
