# Supplementary material for: A mechanistic approach to anti-nociceptive potential of Artemisia macrocephala Jacquem
Source: BMC Complement Altern Med. 2016 May 26;16:141. doi: 10.1186/s12906-016-1114-0 (PMC4881202; doi:10.1186/s12906-016-1114-0)
Supplement: Additional file 1: — Spectra analysis. (DOC 6349 kb) [file 12906_2016_1114_MOESM1_ESM.doc]

This spectroscopic study has been carried out in International Center for Chemical and Biological Sciencs (ICCBS), HEJ, Karachi. Since many samples were given at once, therefore, there is a bit change in the sample name on the spectra and in the manuscript. Therefore, the names, for the purpose of ease are clarified as:

Spectral Ism-4 = Ism-1 in the manuscript

Similarly Ism-6 = Ism-2, Ism-2 = Ism-3 and Ism-8 = Ism-4.

**
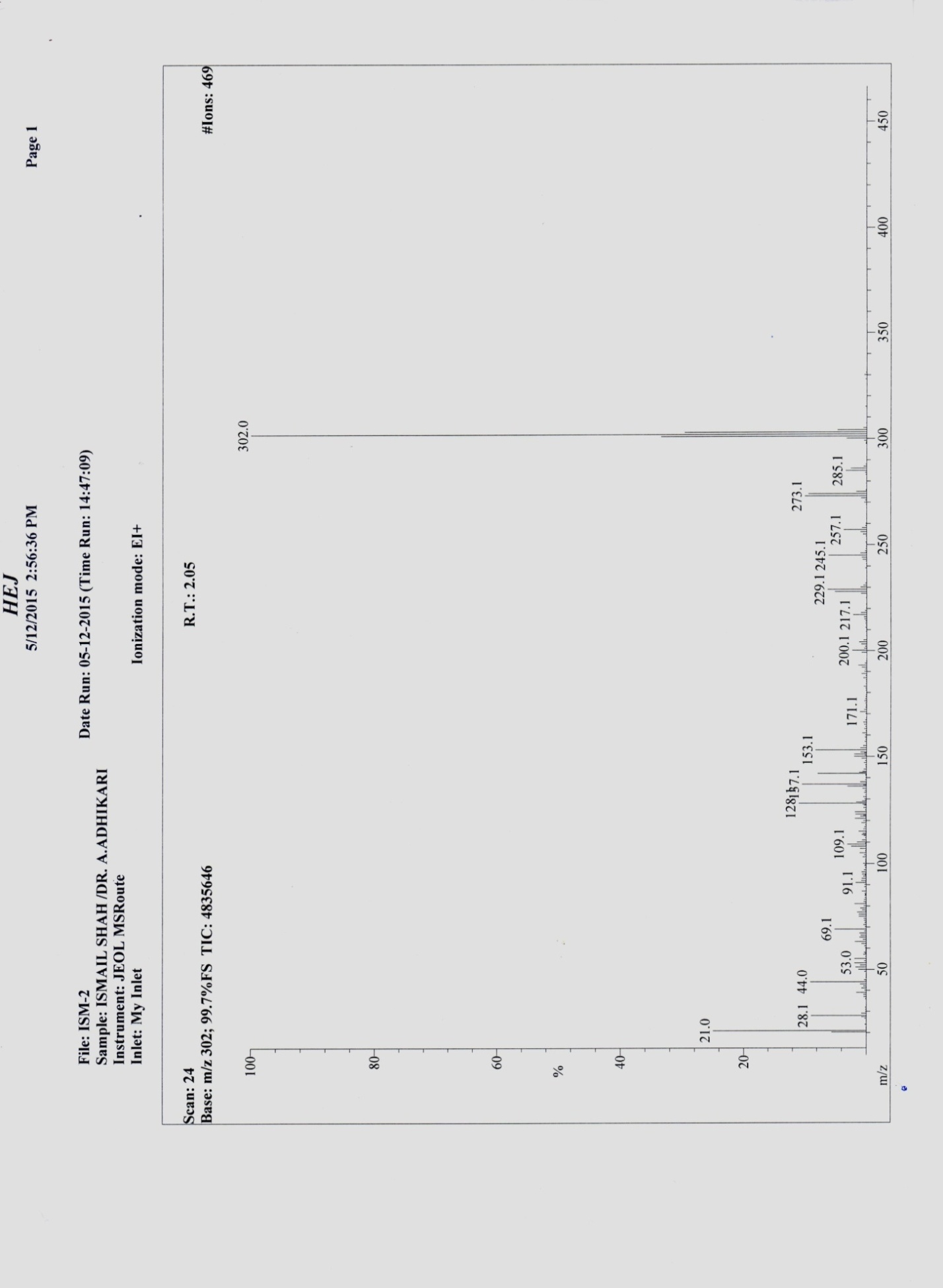
**

**
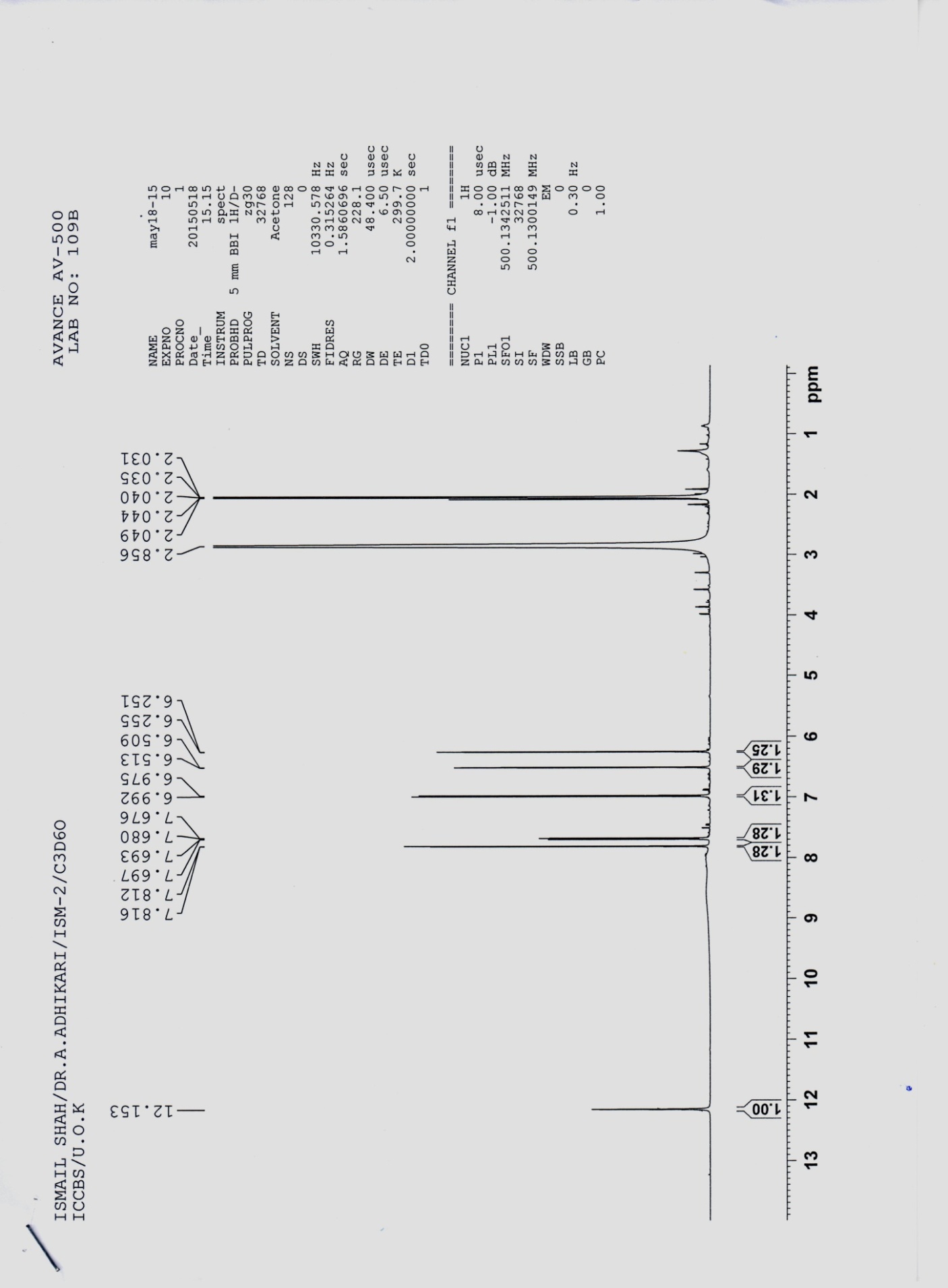
**

**
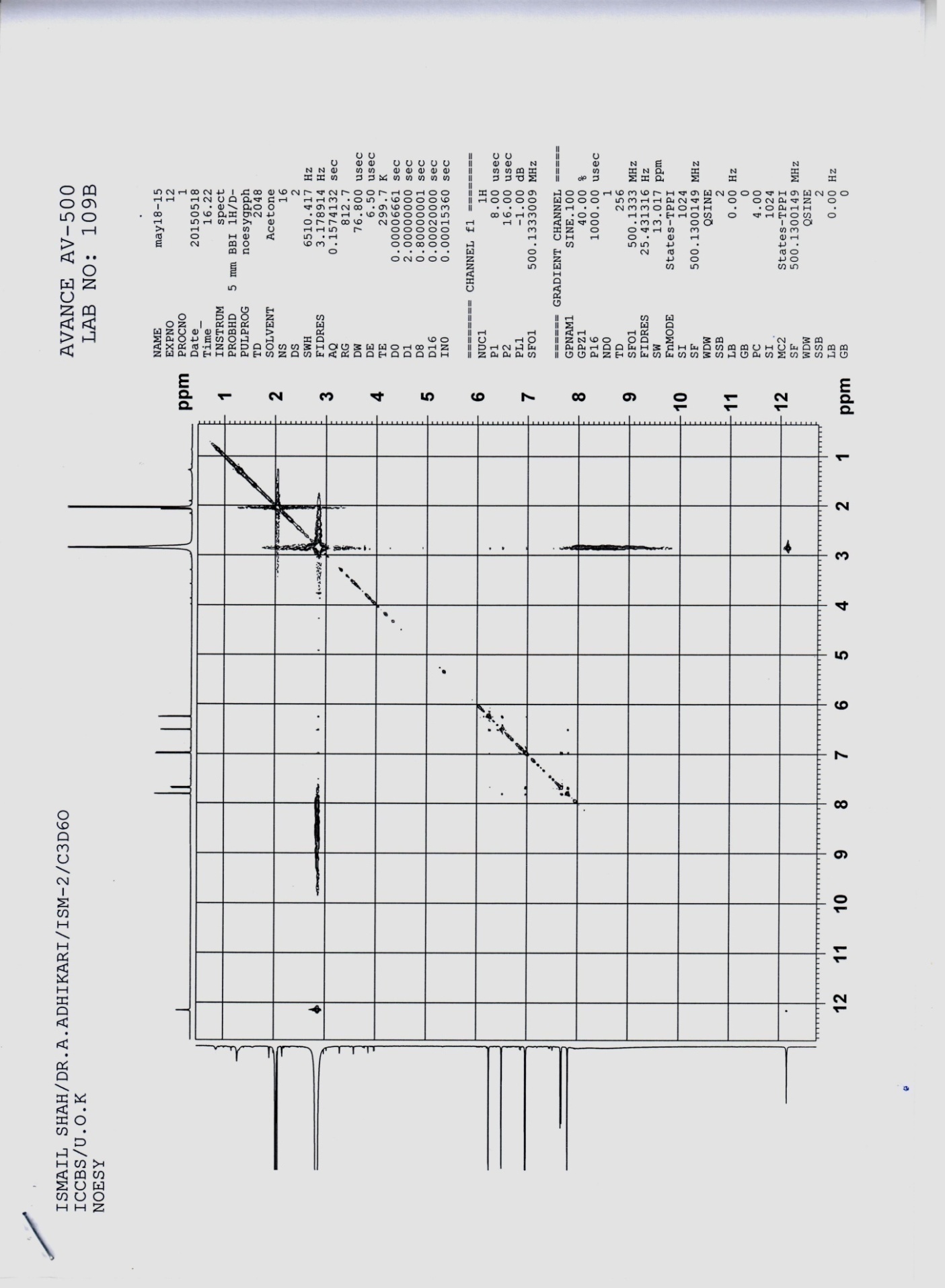
**

**
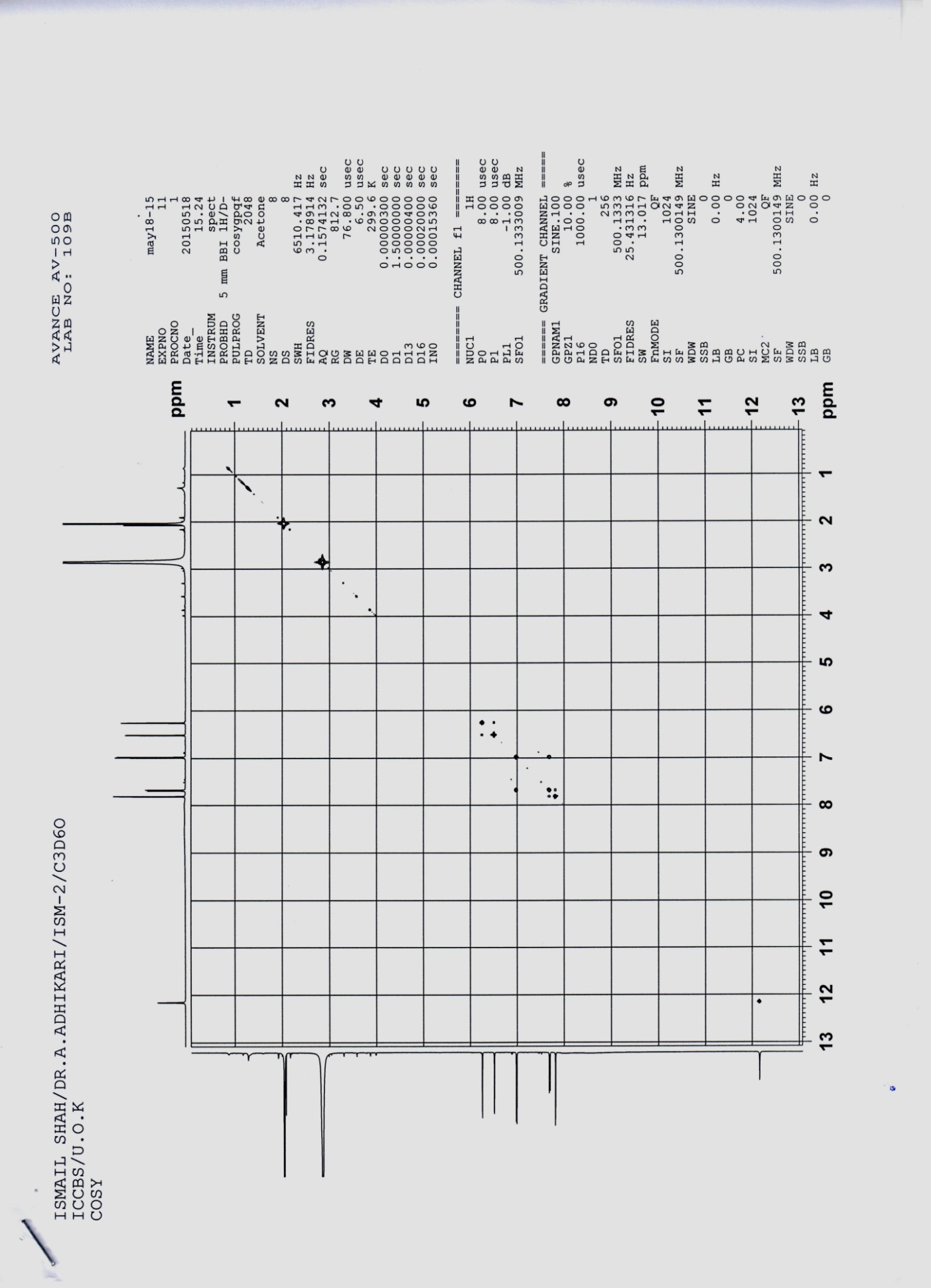
**

**
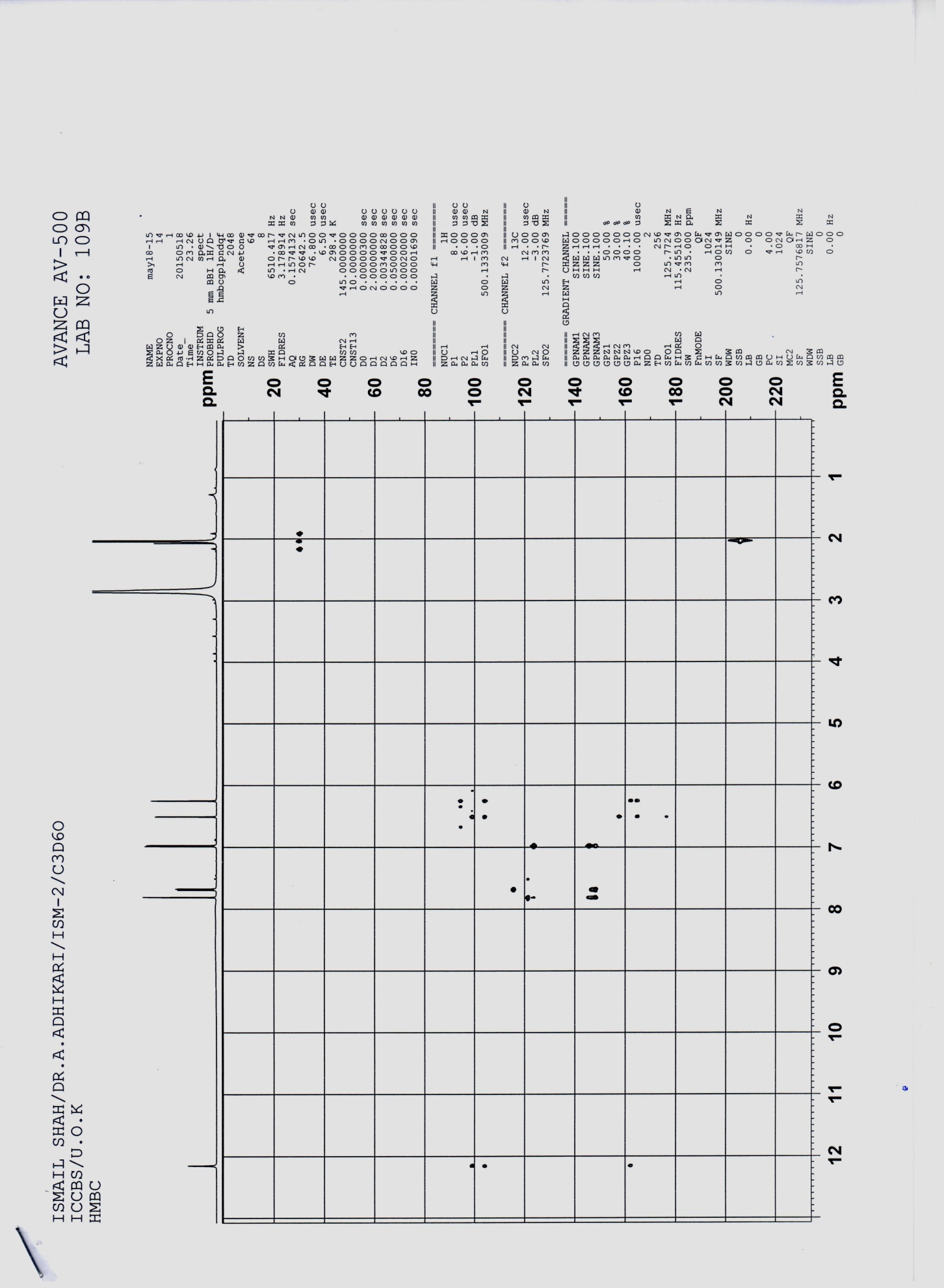
**

**
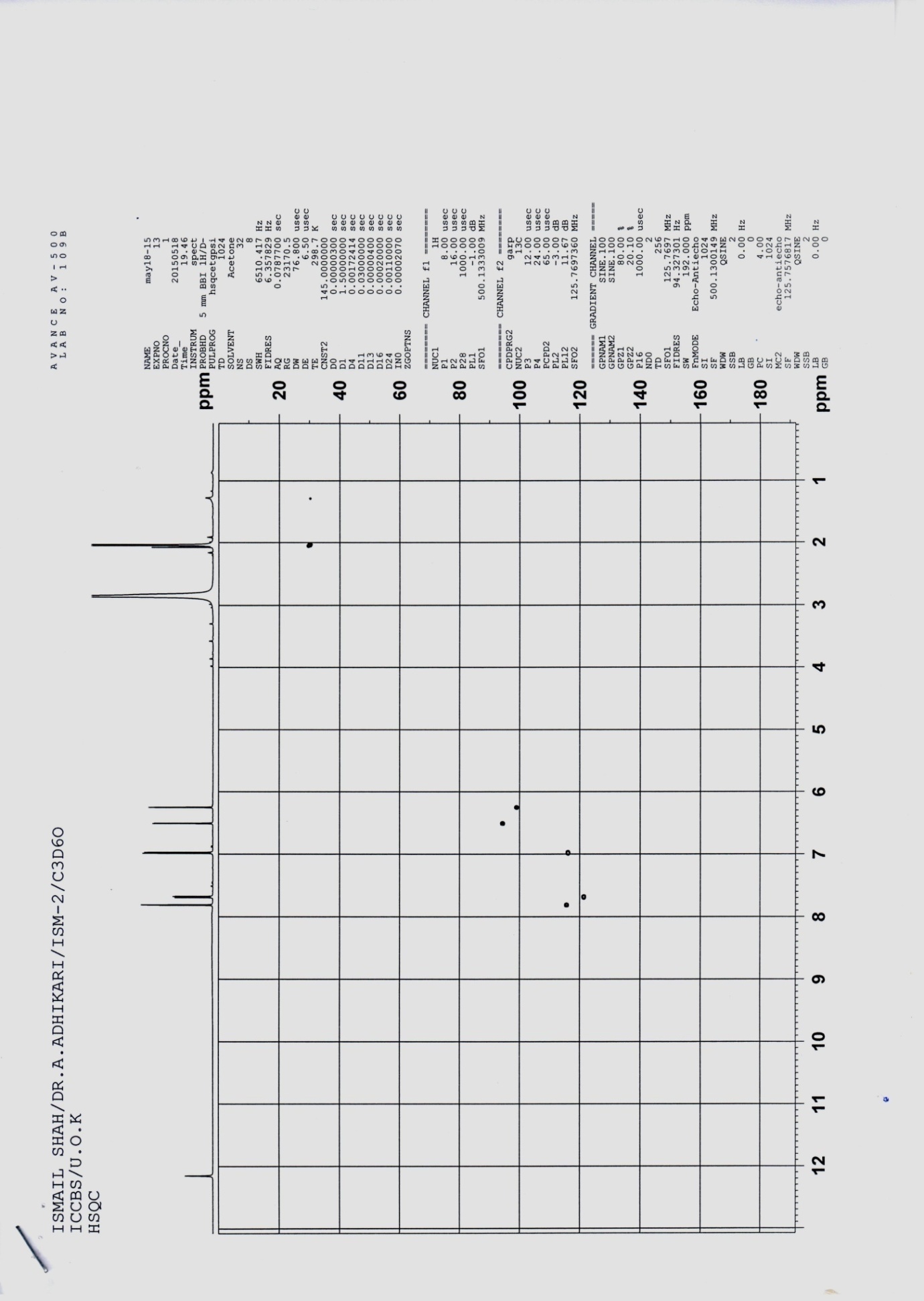
**

**
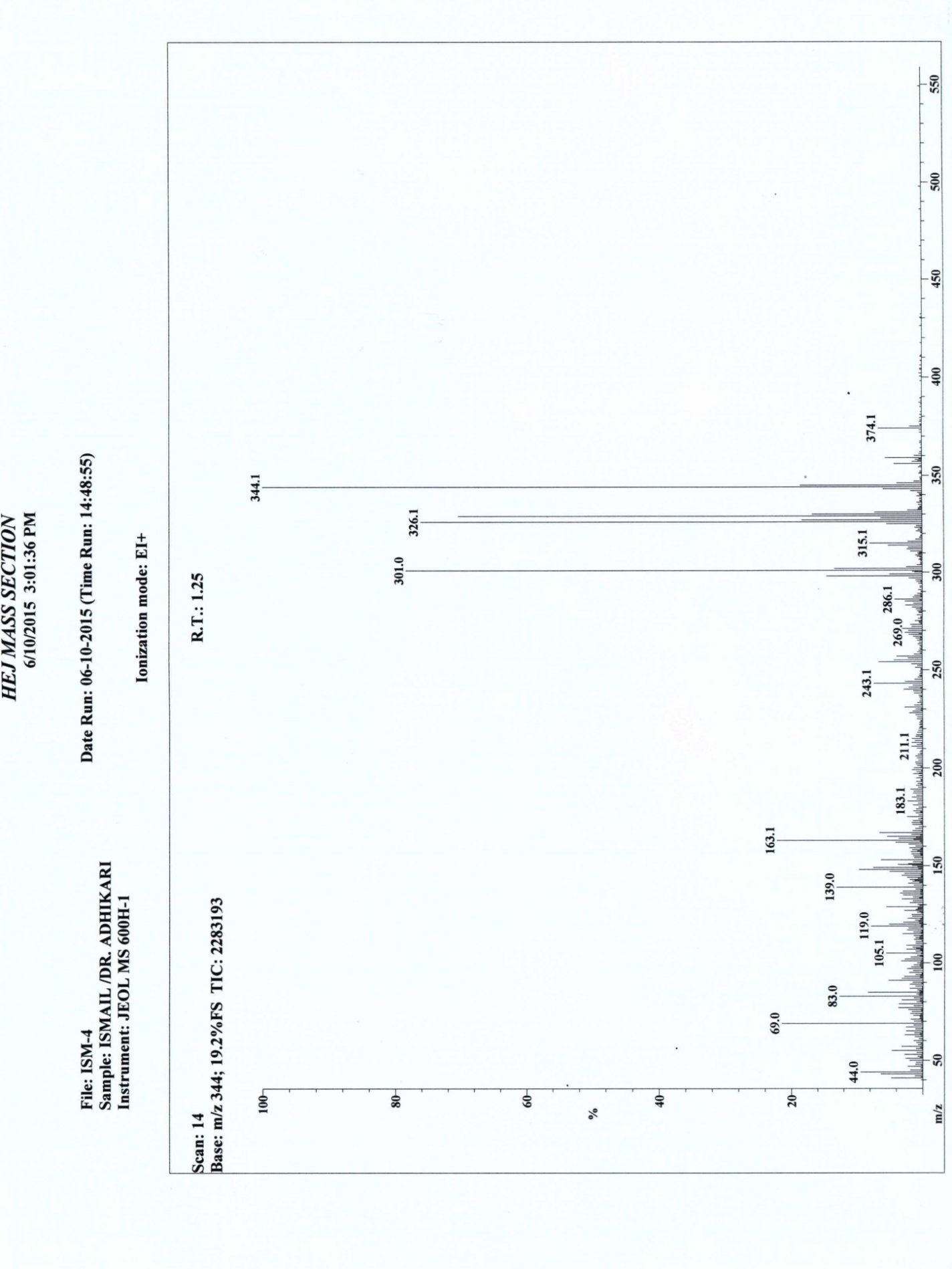
**

**
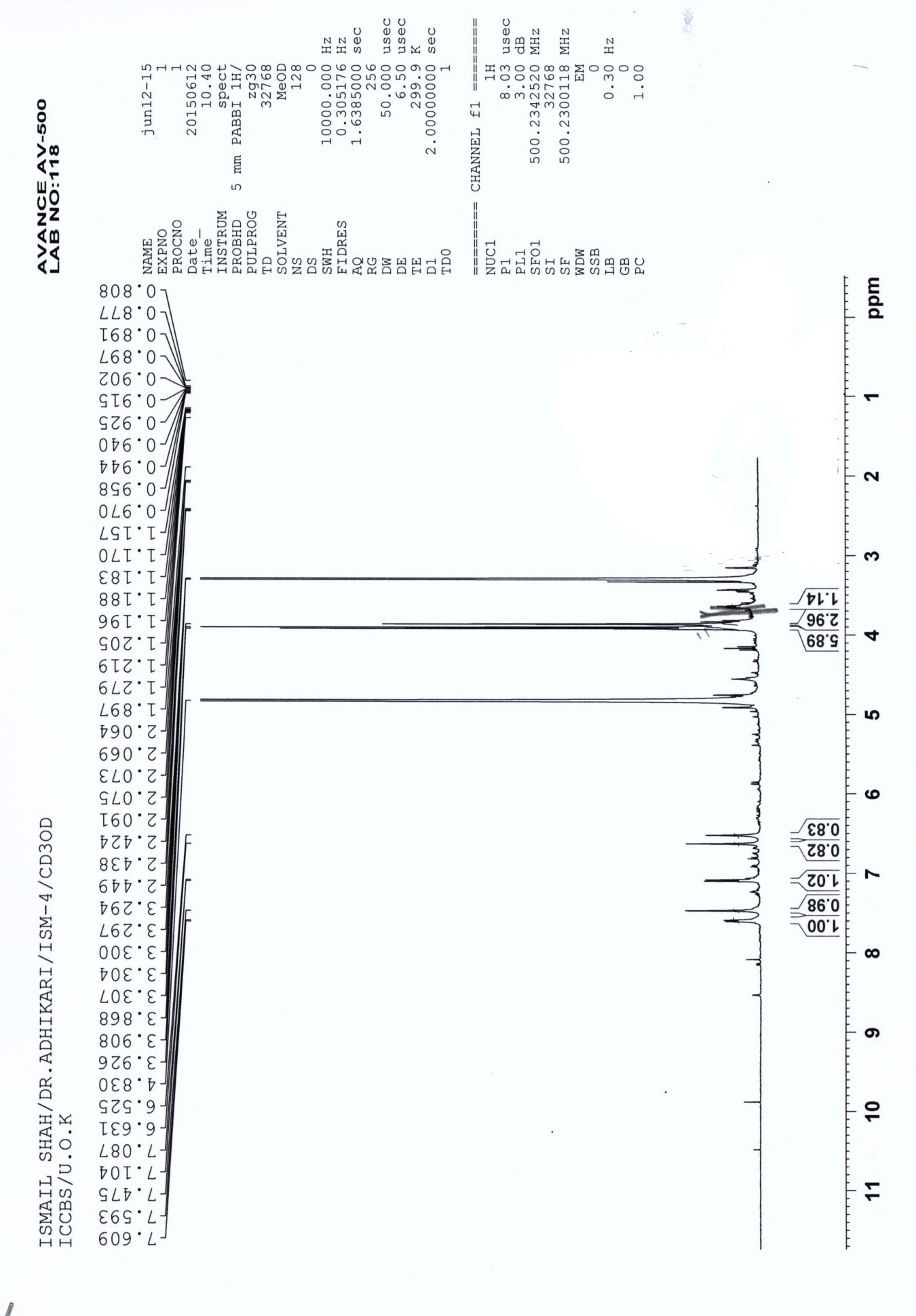
**

**
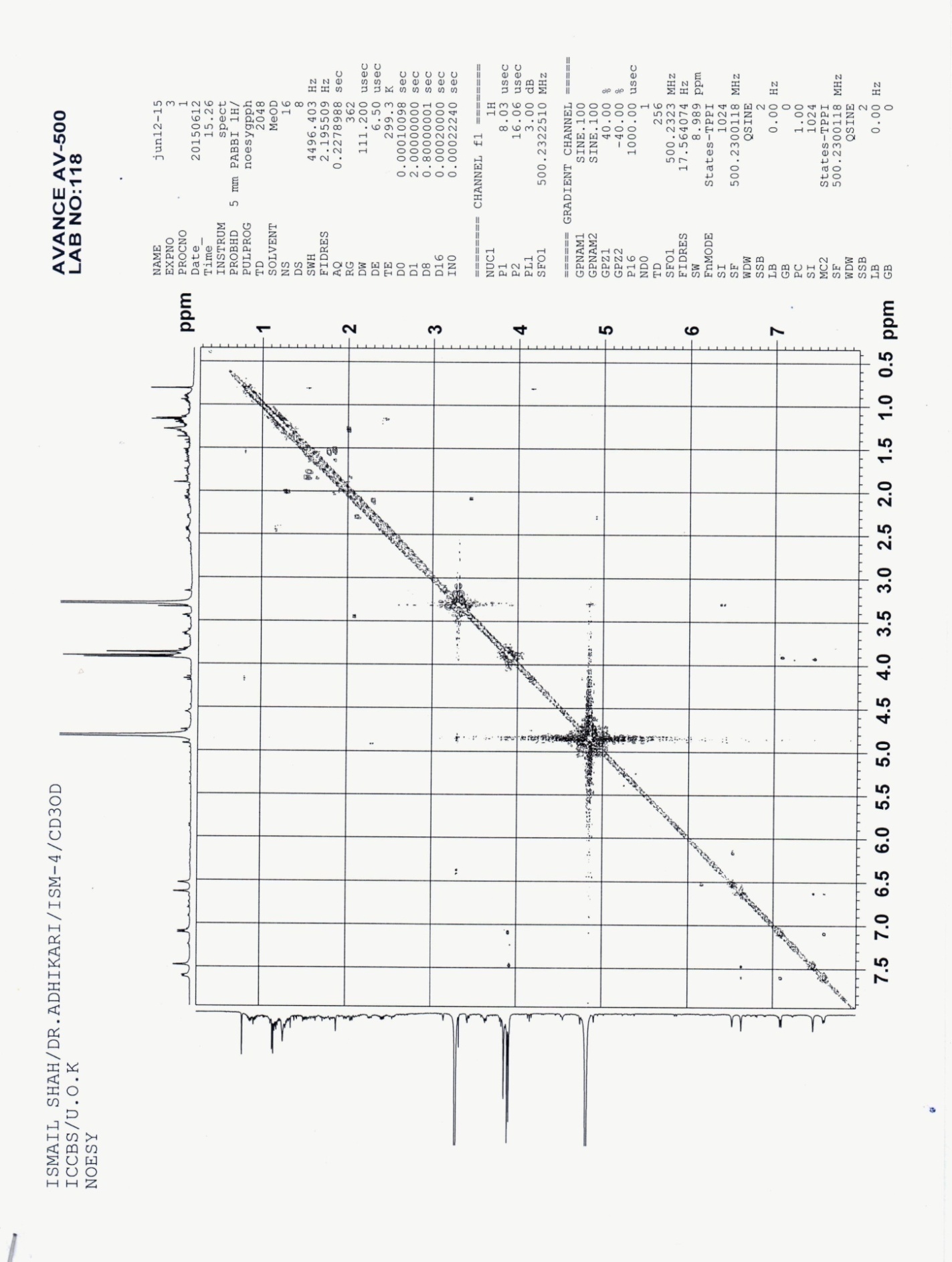
**

**
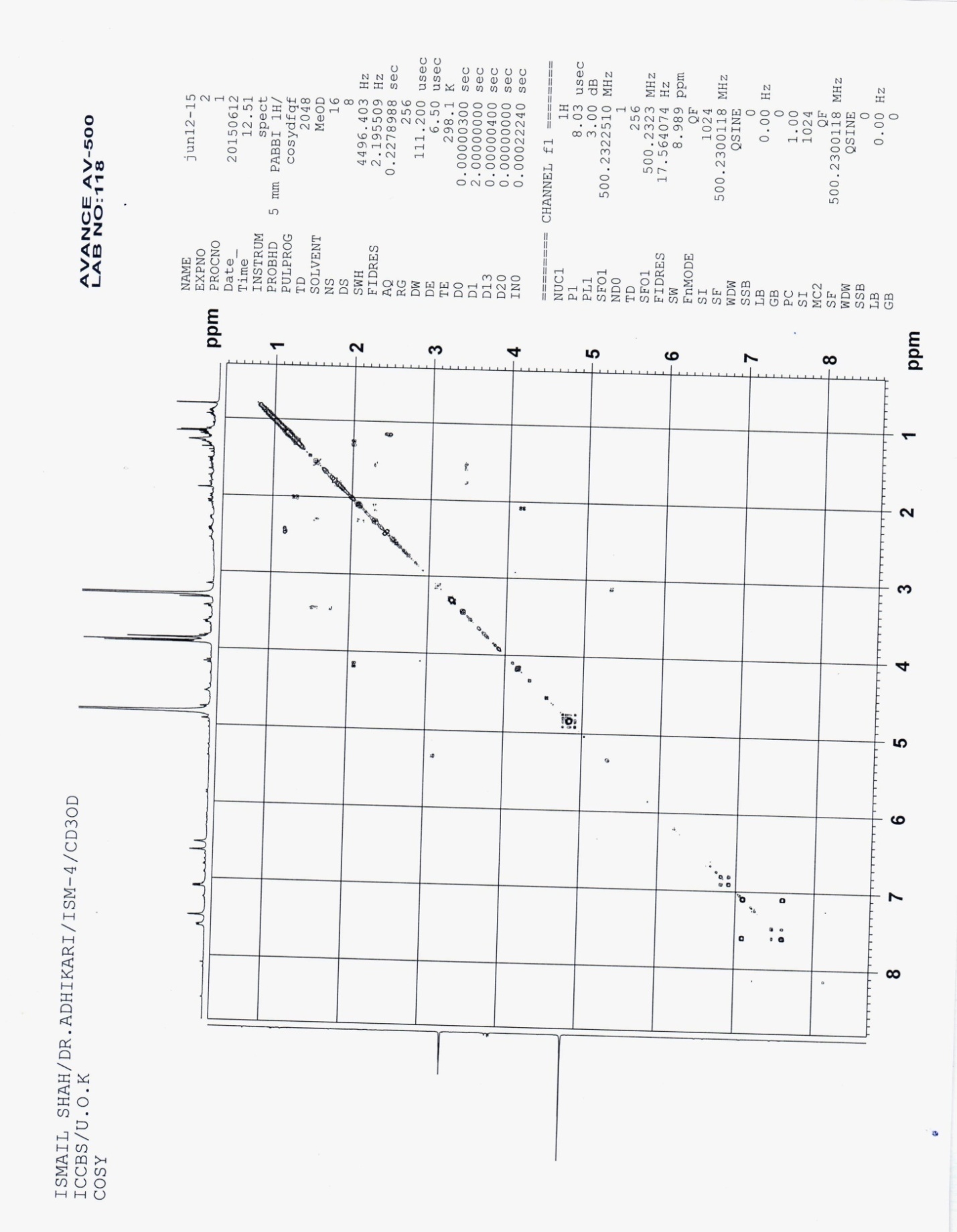
**

**
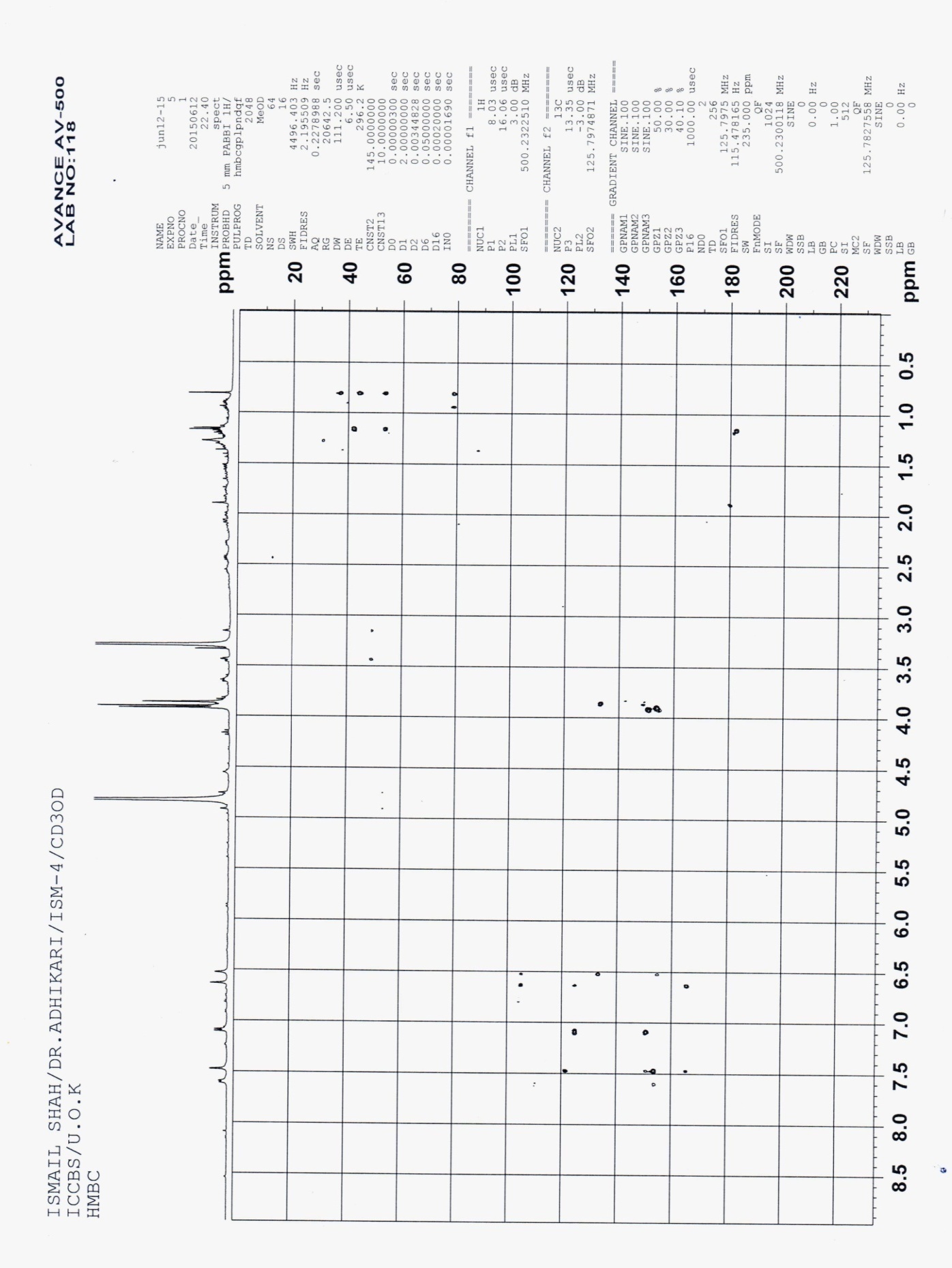
**

**
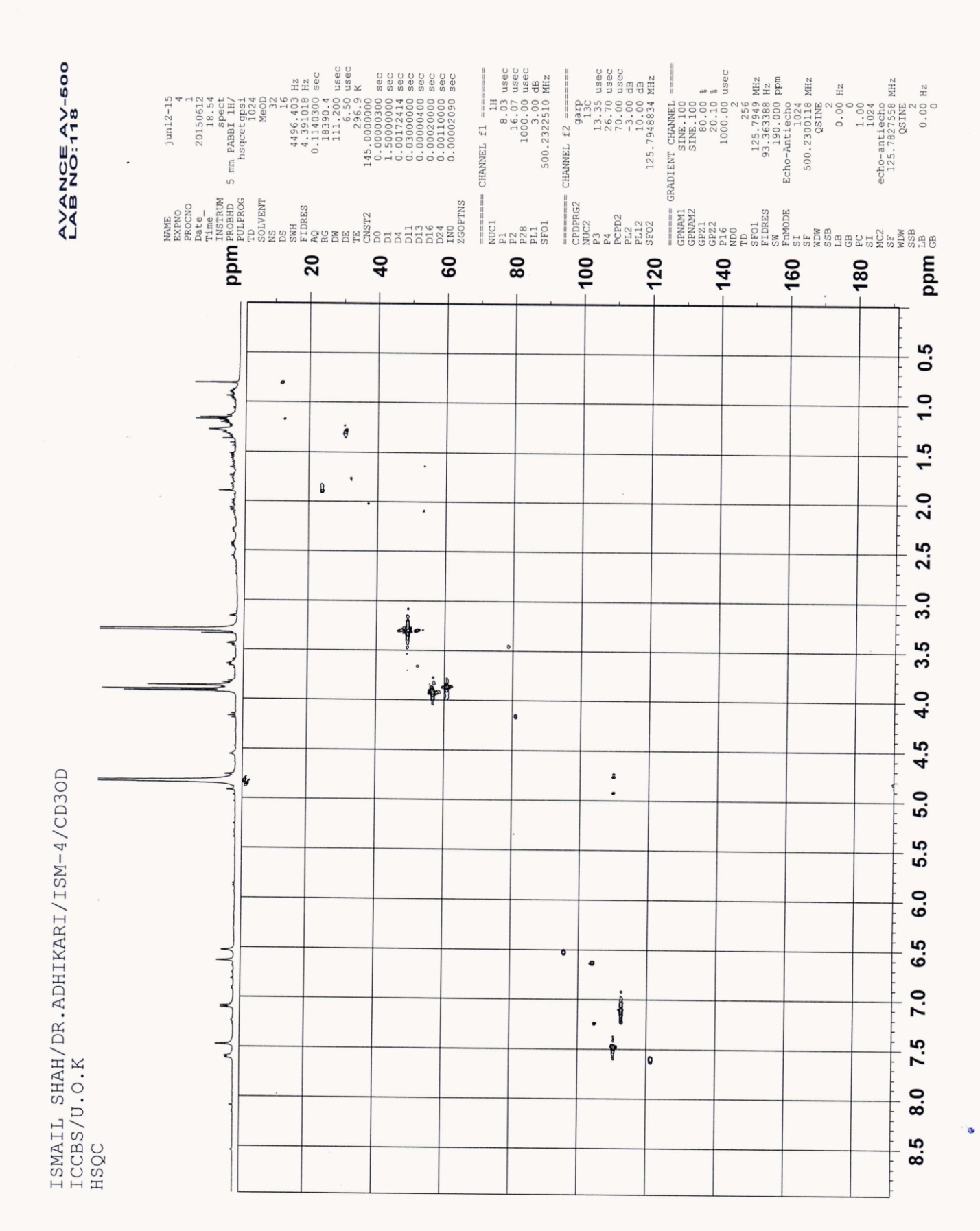
**

**
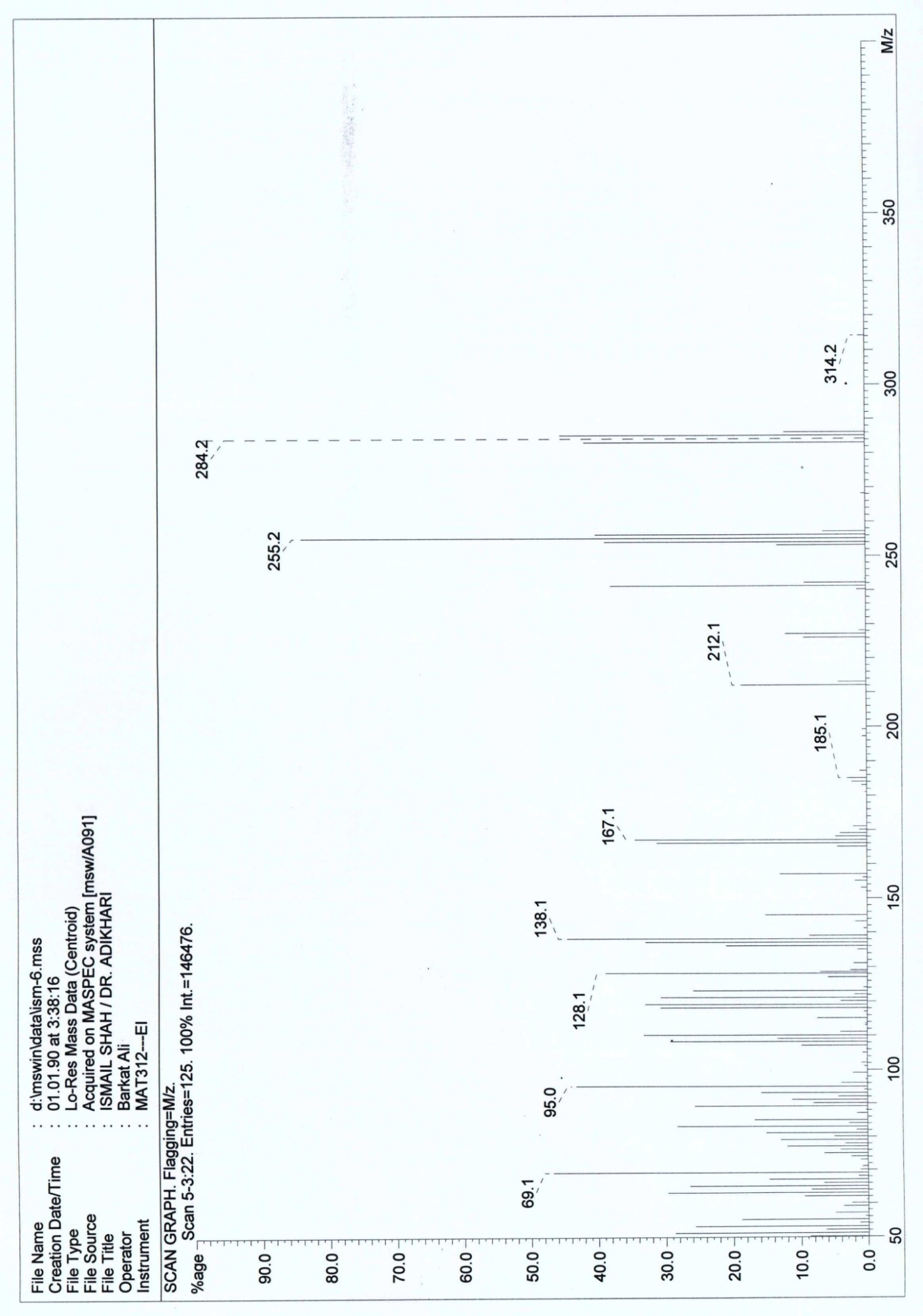
**

**
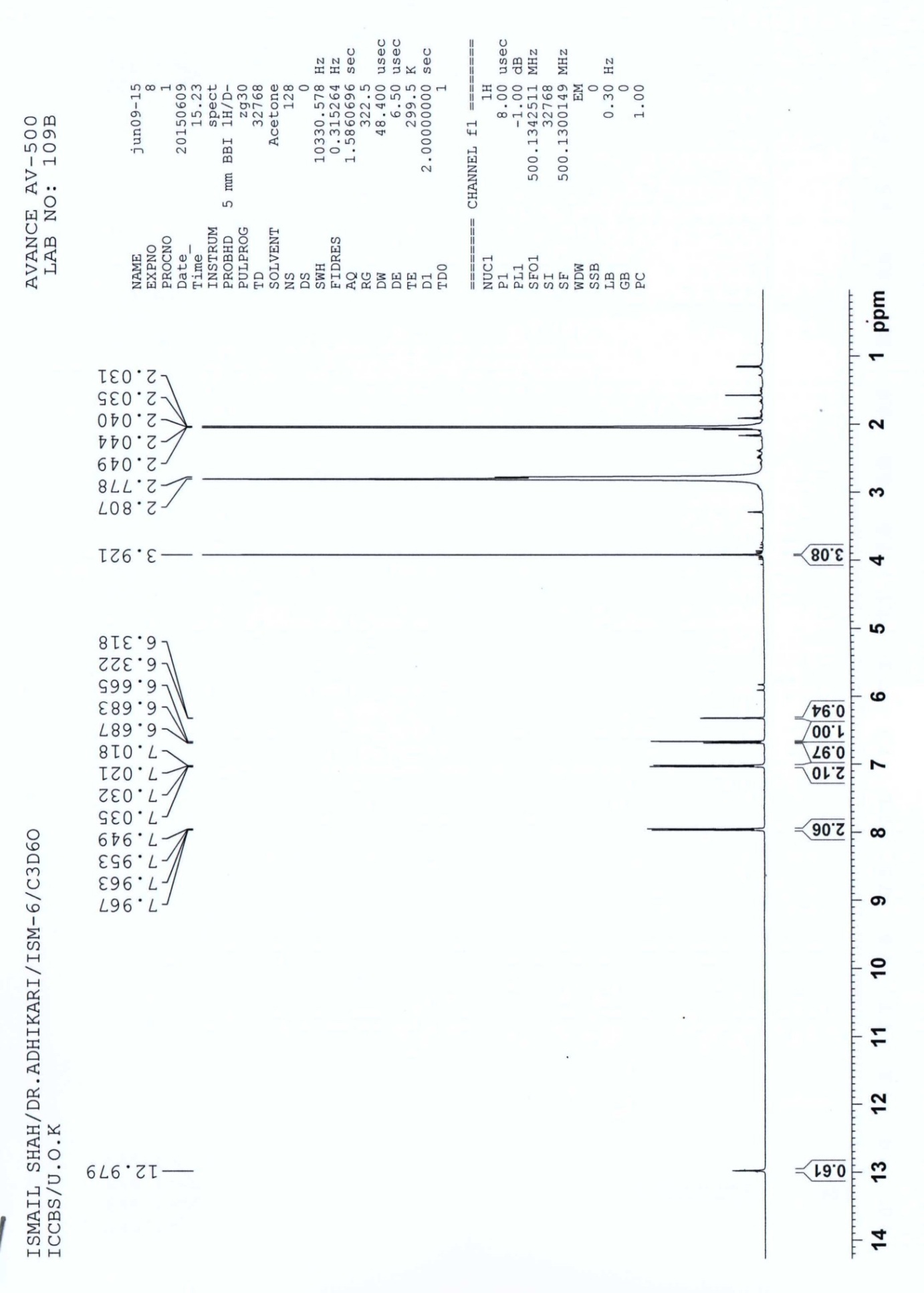
**

**
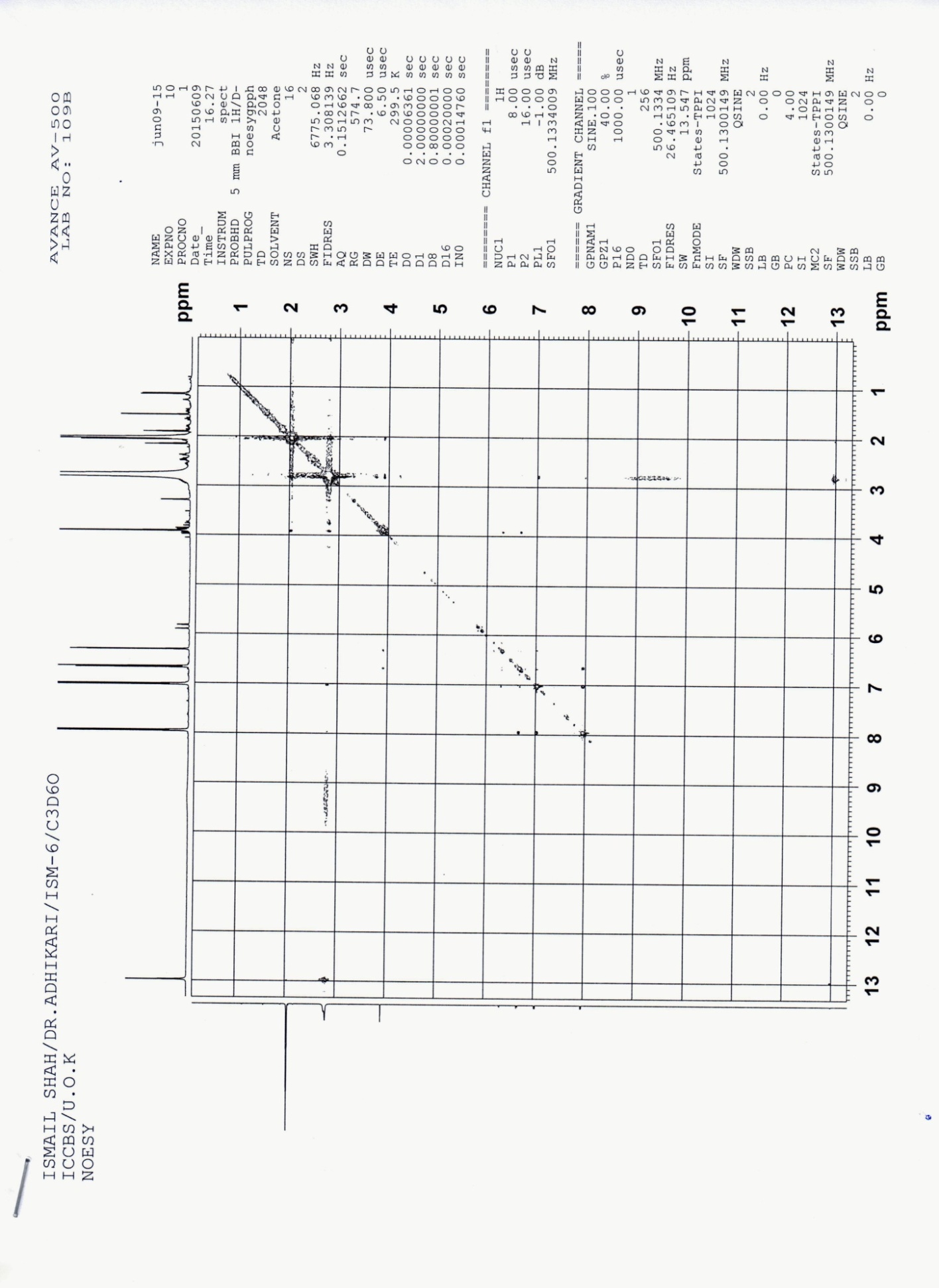
**

**
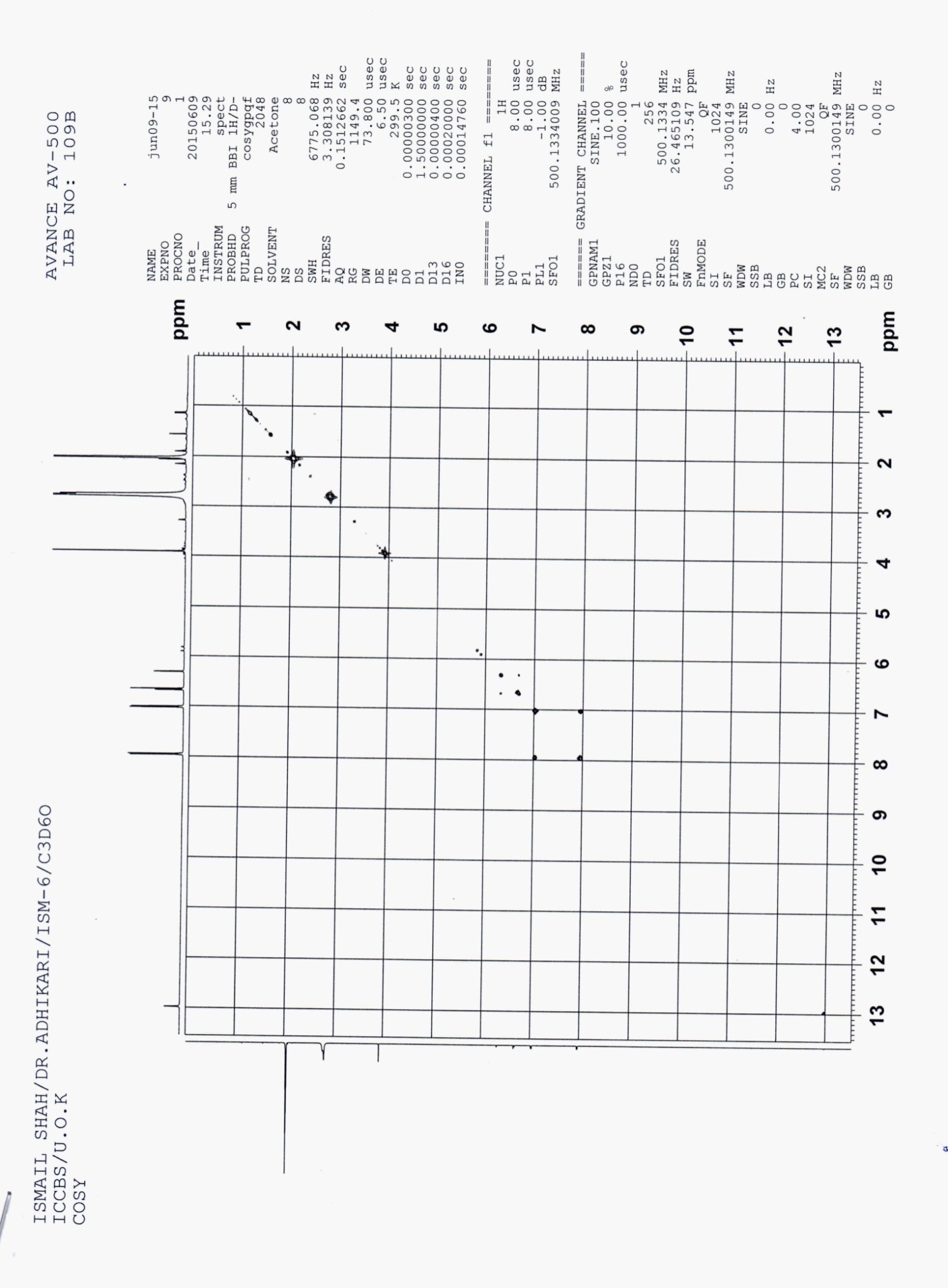
**

**
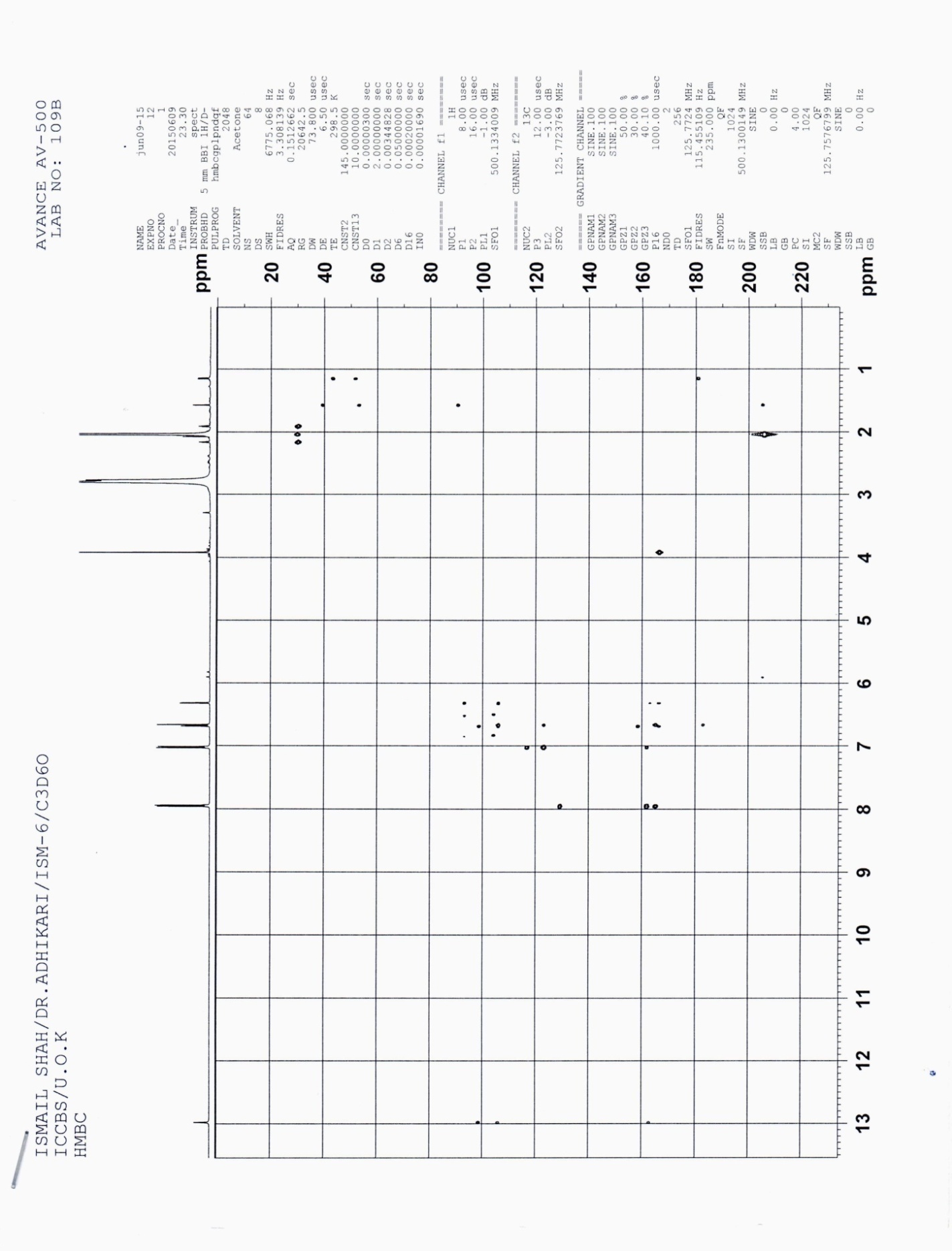
**

**
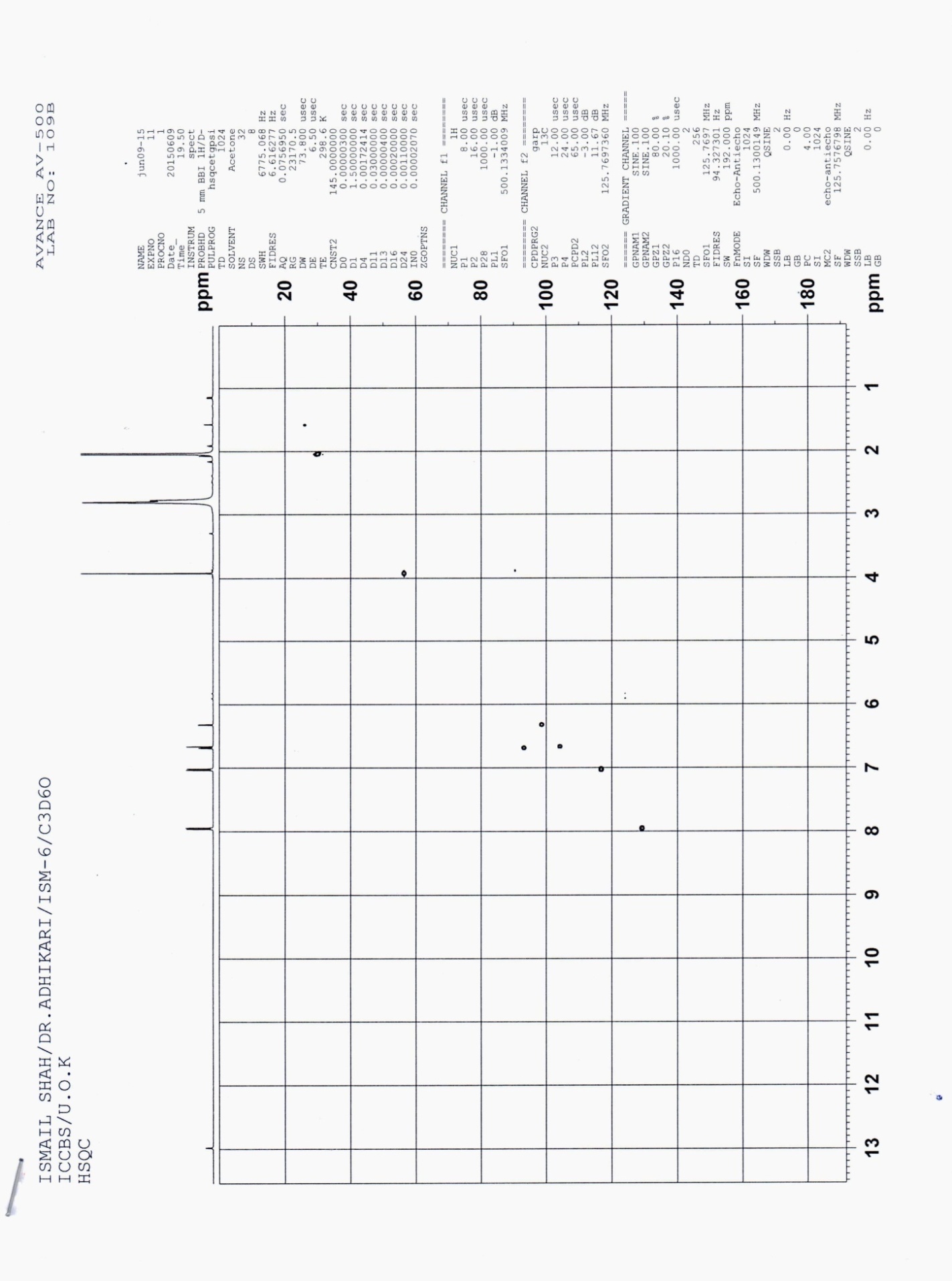
**

**
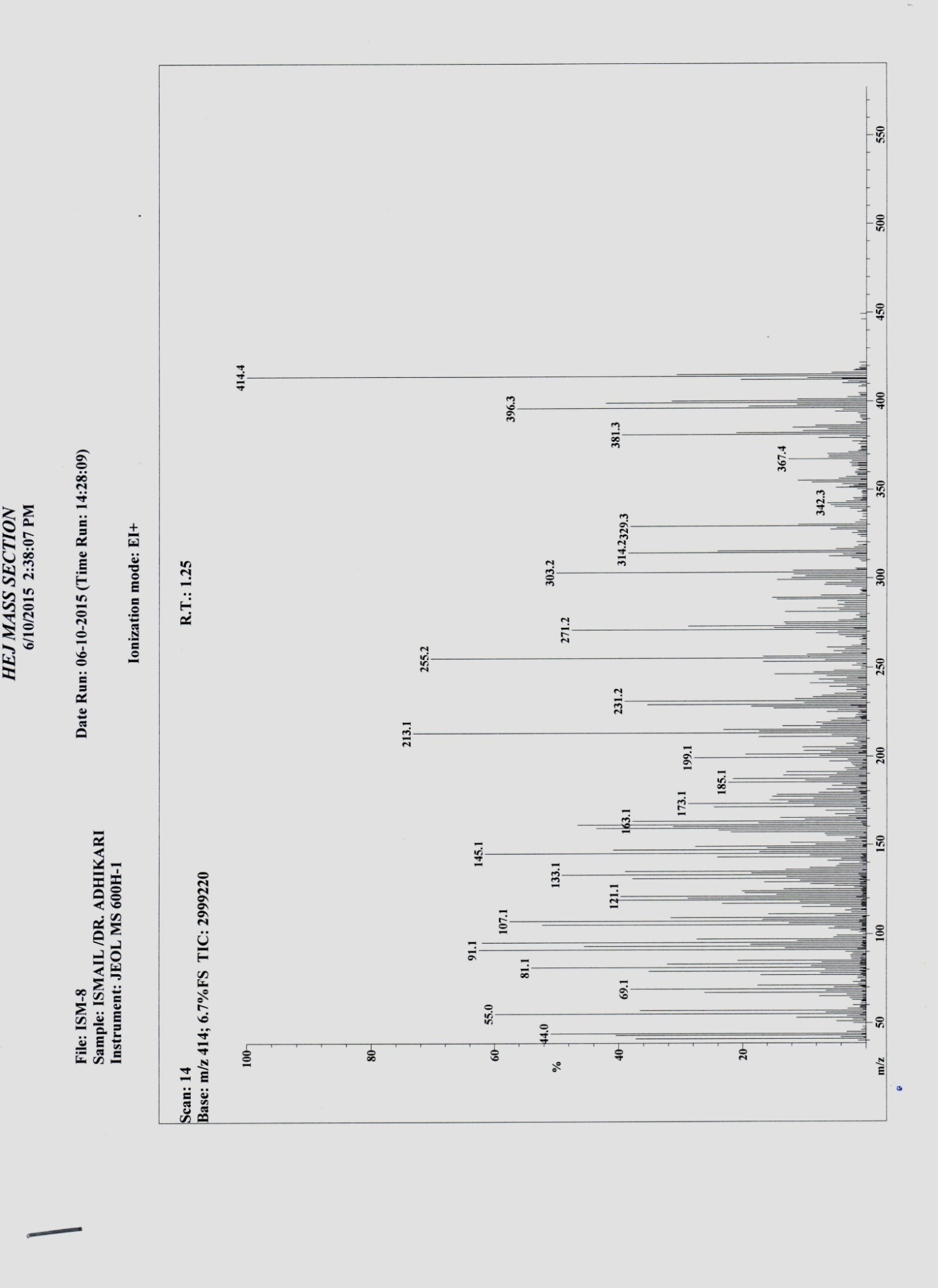
**

**
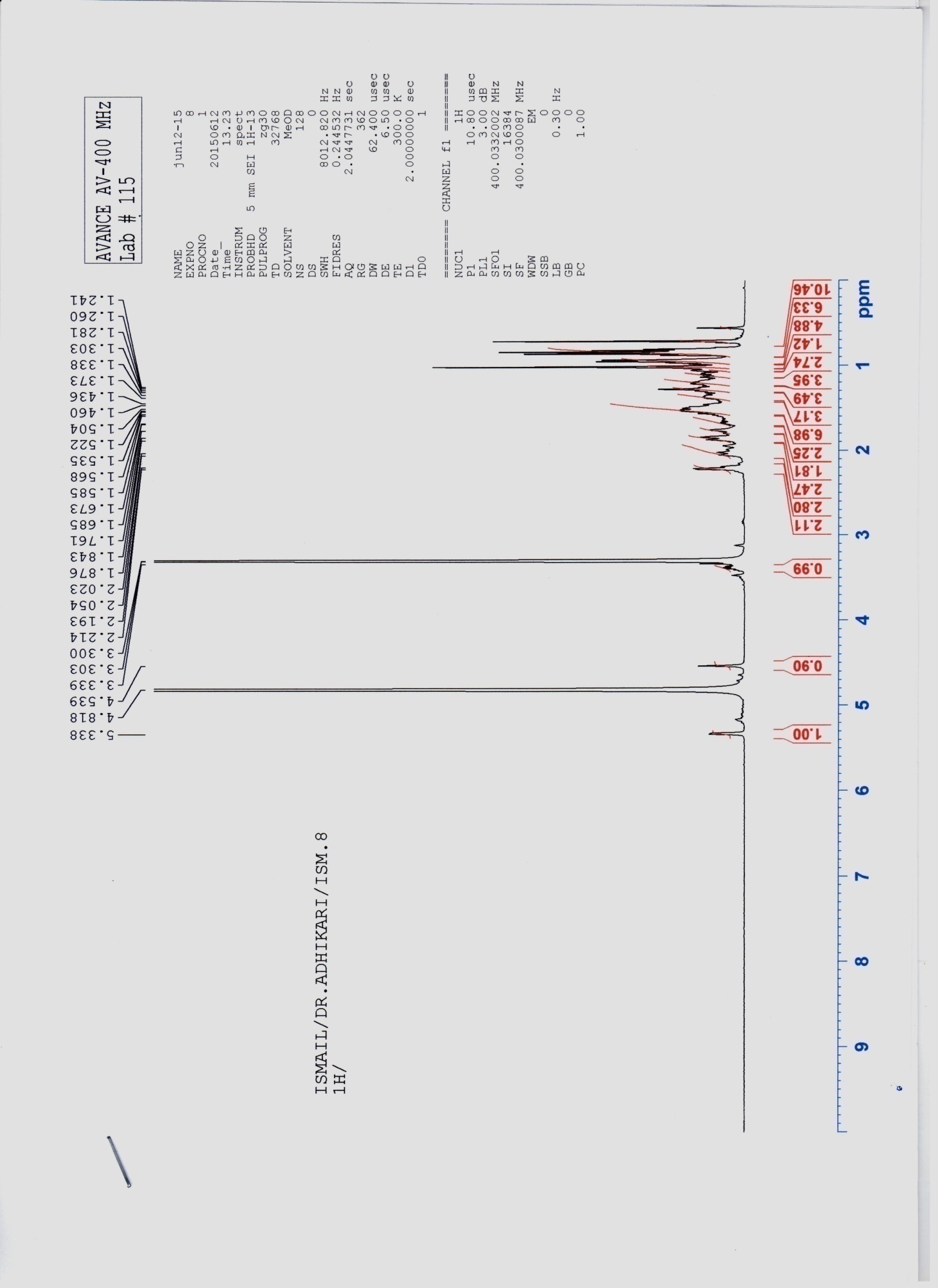
**
